# The benefits of radiological imaging for postoperative orthostatic headache: a case report

**DOI:** 10.1186/s12880-019-0365-x

**Published:** 2019-08-07

**Authors:** Mu-Jung Lee, Chih-Jen Hung

**Affiliations:** 1 Department of Anesthesiology, Shin Kong Wu Ho-Su Memorial Hospital, No.95, Wenchang Rd., Shilin Dist, Taipei City, 111 Taiwan, Republic of China; 2 Department of Anesthesiology, Taichung Veterans General Hospital, No.1650, Sec. 4, Taiwan Blvd., Xitun Dist, Taichung City, 407 Taiwan, Republic of China

**Keywords:** Case report, Headache, Intracranial hypotension, Post-dural puncture headache

## Abstract

**Background:**

Traditionally, the diagnosis of post-dural puncture headache (PDPH) relied upon the patient’s history regarding dural puncture and symptoms, such as orthostatic headache. However, such evidence may not always be reliable or specific. We report an unexpected diagnosis with spontaneous intracranial hypotension (SIH), which was confirmed upon examination of Magnetic Resonance (MR) images in a patient who was initially suspected to have PDPH because he had recently undergone a uncertain dural puncture.

**Case presentation:**

A 45-year-old man had undergone a thoracic epidural catheter insertion for perioperative analgesia prior to general anesthesia induction. Due to intermittent dripping of fluid while the epidural needle was being advanced, a dural puncture was suspected. The patient complained of an orthostatic headache after recovery from surgery, therefore a PDPH was suspected. MR images revealed signs of SIH: dural sinus engorgement, contrast enhancement along the neural sleeves of the left C6–7, bilateral C7-T1, T1–2, T2–3, T3–4, T4–5, and T5–6. Computed tomography-guided epidural blood patching (EBP) was performed the following day, with the patient experiencing immediate relief of the headache.

**Conclusion:**

The benefits of radiological imaging in this case included confirming the correct diagnosis, guiding the accurate level and proper approach of EBP, distinguishing the epidural space from the intrathecal space, and ultimately increasing the likelihood of successful EBP.

## Background

Spontaneous intracranial hypotension (SIH), which is characterized by spontaneous cerebrospinal fluid (CSF) leakage, has an incidence of 5 cases per 100,000 people each year [1]. Its most common symptom is an orthostatic headache. Patients with a history of trauma or congenital connective disease have fragile dura, and thus are more susceptible to SIH. Cases of SIH after epidural injection have been reported sporadically in the literature [2, 3]. The diagnosis of SIH usually relies upon images or a patient’s clinical symptoms. Epidural blood patch (EBP) application is an established treatment for SIH. EBP application involves the injection of autologous blood into the epidural space to stop CSF leakage. Herein, we report the case of a patient whose initial impression of post-dural puncture headache (PDPH) was changed to SIH after magnetic resonance (MR) images were reviewed. The CSF leakage locations were far from the previous puncture sites used for the epidural catheterization. A massive CSF leak in this case would have made EBP difficult when attempting a blind technique. Computed tomography (CT)-guided EBP was therefore perform, and the patient had immediate relief of the headache.

## Case presentation

This 45-year-old male denied having any systemic diseases or any surgical history prior to admission to our center for a thoracoscopic lung segmentectomy for cryptococcus infection. Epidural catheter insertion for perioperative analgesia was performed prior to the induction of general anesthesia without sedation. The first attempt of epidural catheter insertion was performed with a Tuohy needle using the loss of resistance to air technique at the T7–8 interspinous space. However, an intermittent fluid drip was discovered as the Tuohy needle was advanced. A dural puncture was suspected, and the procedure was then repeated through the T8–9 interspinous space. However, an intermittent fluid drip was found once again as the Tuohy needle was advanced, so the procedure was abandoned. Both general anesthesia and the surgery were performed uneventfully.

The patient complained of both headache and dizziness when he sat up in the ward after the operation. The symptoms were attenuated when he was in a supine position. PDPH was suspected and hydration and analgesics were prescribed. By the fifth postoperative day, the patient was unable to either stand or walk for more than 5 min without recurrence of the headache. A neurologist was consulted, and meningitis was considered as a differential diagnosis. Brain and whole spine MR imaging were ordered for further evaluation.

**Fig. 1:**
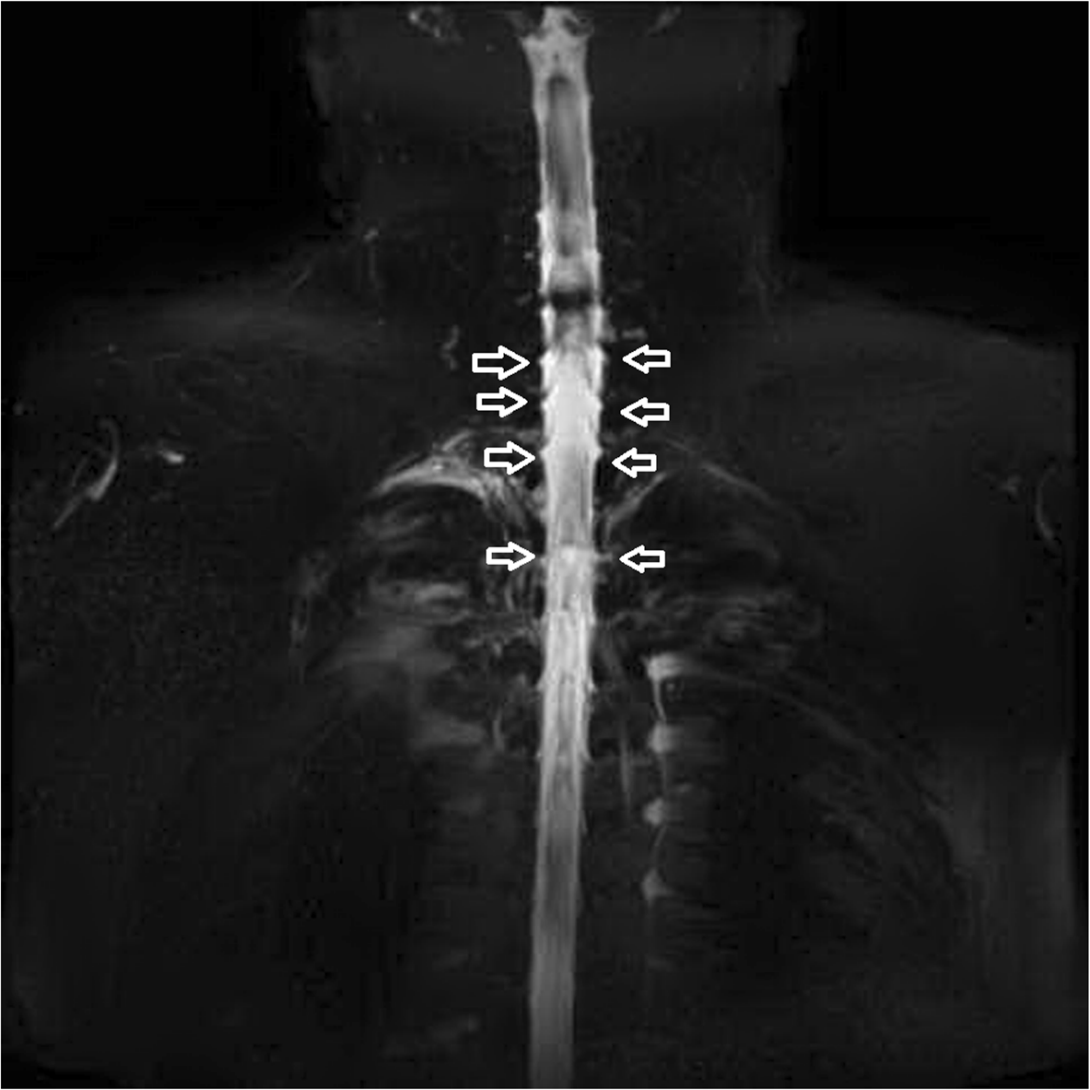
Many CSF leak lesions of lower C-and upper T spine were found using thin-slice axial multi-planar reconstruction MRM in T2-weighted three-dimensional sampling perfection with application-optimized contrasts using different flip-angle evolutions pulse sequence. Arrow: CSF leak lesions

**Fig. 2:**
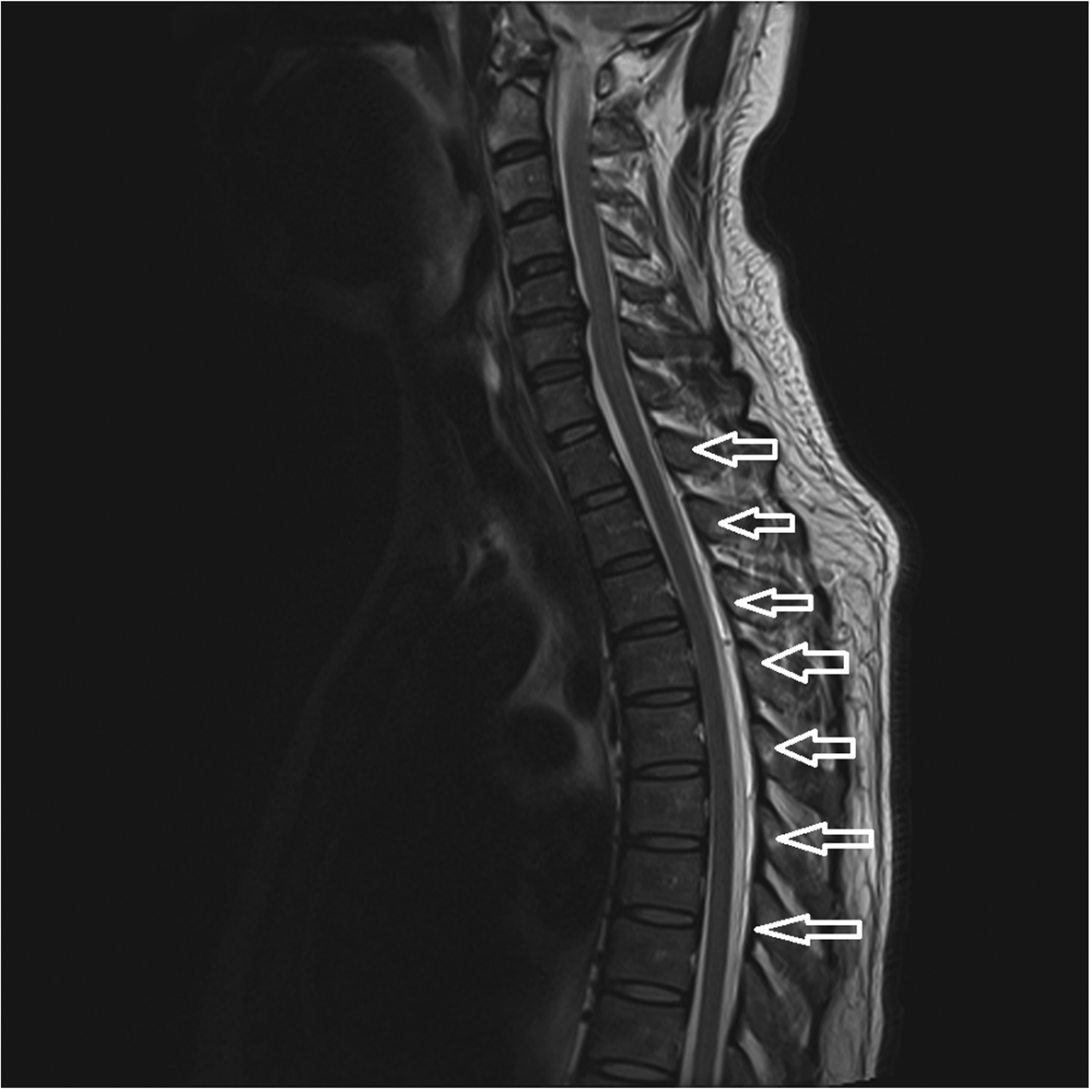
T2-weighed MR image revealed epidural fluid accumulation at the dorsal aspect of the T1–9 spinal canal. Arrow: epidural fluid accumulation

**Fig. 3:**
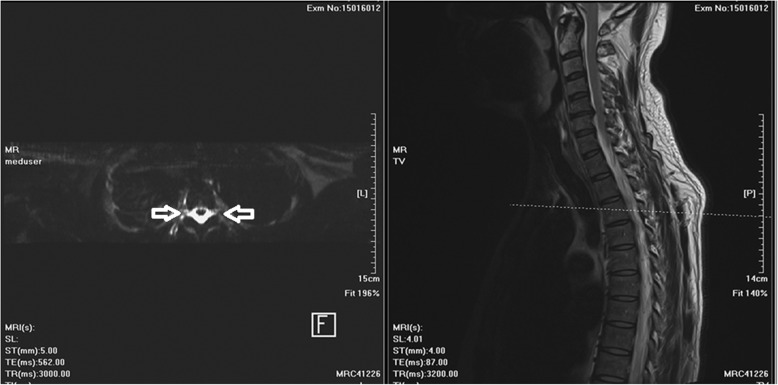
T2-weighed MR image reveals contrast enhancement along the bilateral neural sleeves of T2-T3. The lesion reveals a high signal intensity stripe with the length less than the width of the thecal sac. According to the typing system proposed by Chen et al. [4], a Type C lesion was defined at T2-T3. Arrow: CSF leak lesions

**Fig. 4:**
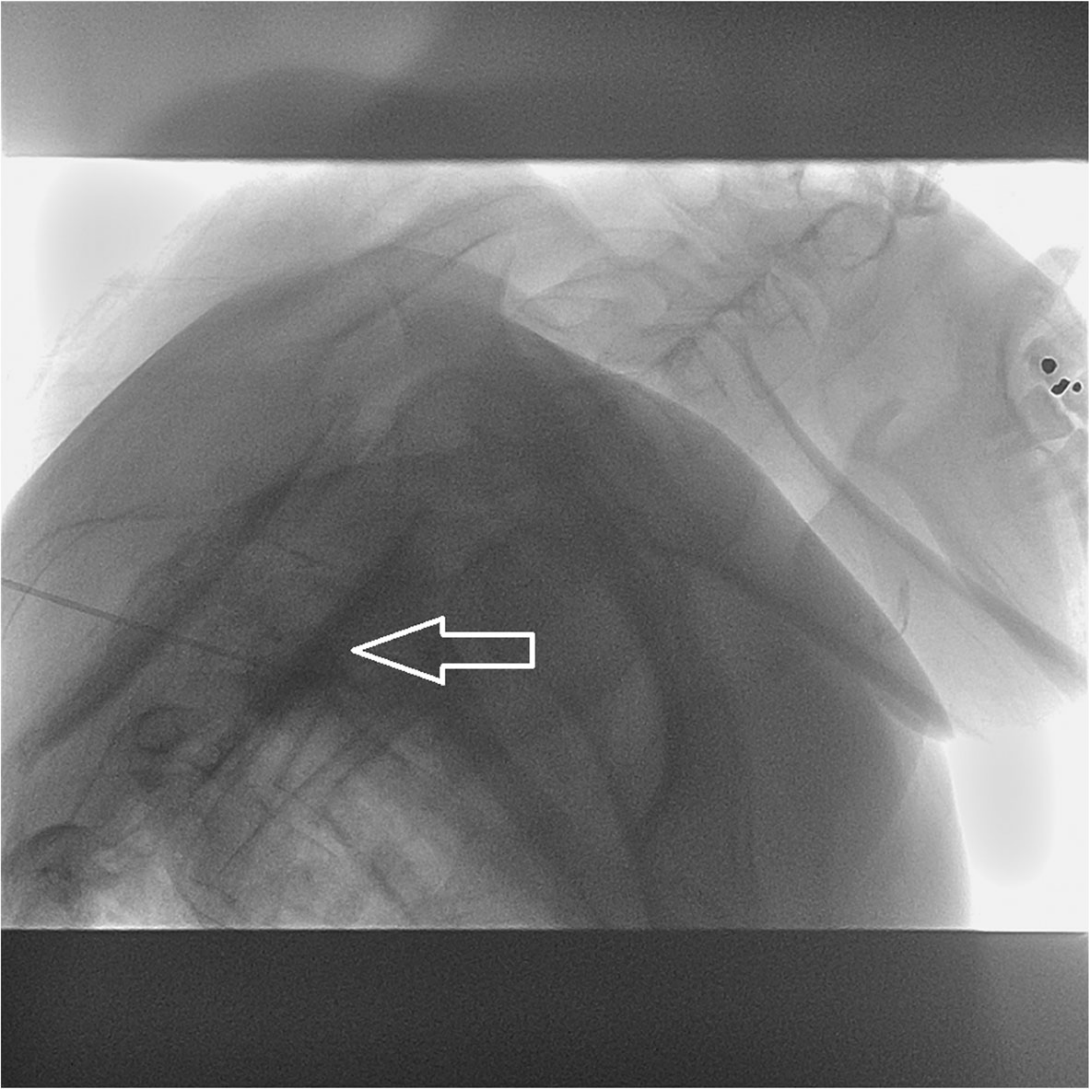
Touhy needle position was confirmed by contrast injection under a fluoroscope at level of T2–3.Arrow: contrast injected

**Fig. 5:**
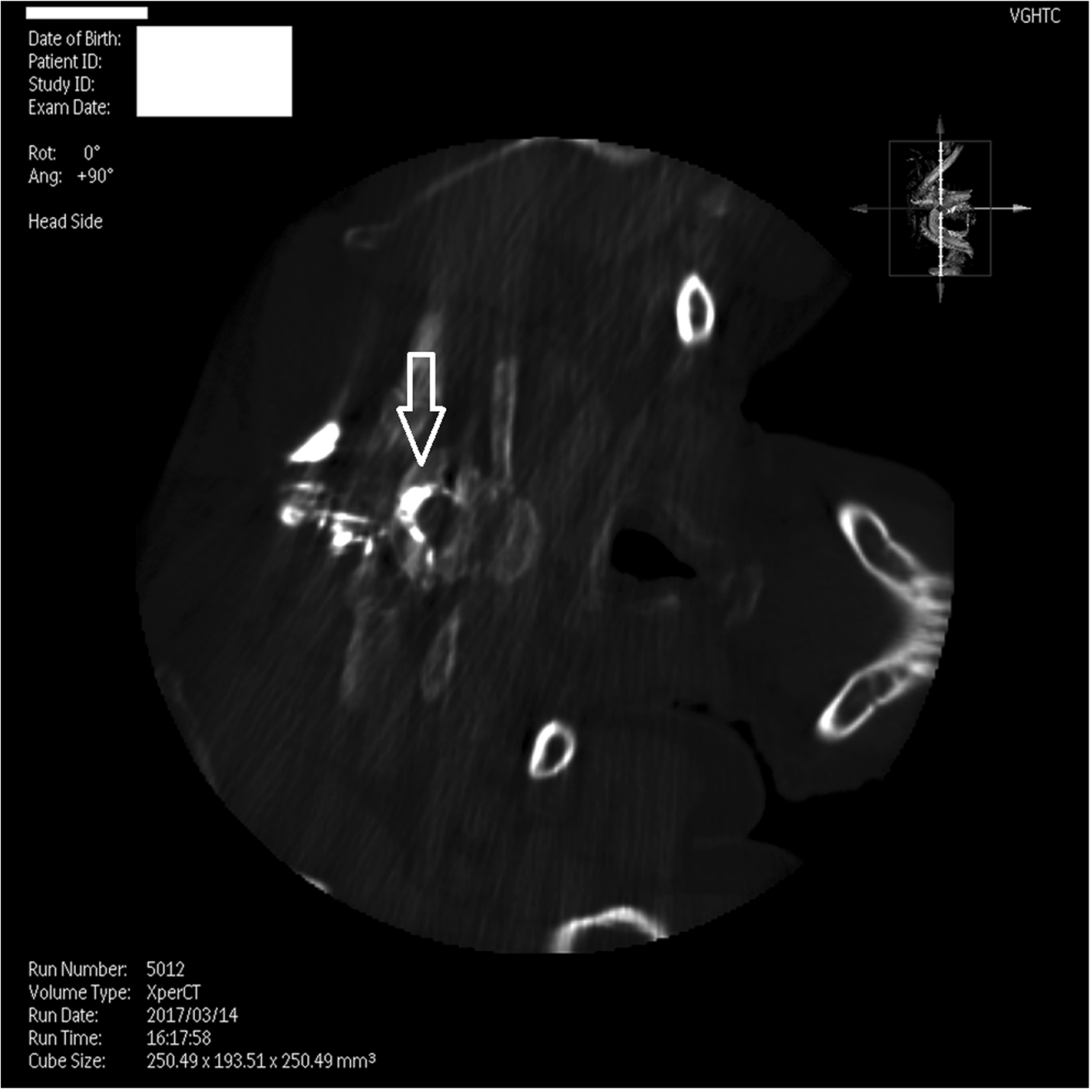
CT-epidurography. Arrow: contrast enhanced at the T2–3 epidural space

The MR images revealed dural sinus engorgement, contrast enhancement along the neural sleeves of the left C6–7, as well as bilateral C7-T1, T1–2, T2–3, T3–4, T4–5, and T5–6. High signal intensity stripes with a length less than the width of the thecal sac were found, and type C lesions were defined [4]. MR myelography (MRM) depicted the lesions causing CSF leakage along the C-T spines (Fig. 1). Epidural fluid accumulations at the dorsal aspect of the T1–9 spinal canal were observed (Fig. 2). The diagnosis of SIH was made, and EBP treatment was arranged. CT-guided EBP was chosen because a blind technique might have made it difficult to distinguish the epidural space from the intrathecal space. For the EBP injection, the C7-T1 interspinous space was not chosen because the space was not obviously palpable. The approach from the T1–2 interspinous space was tried, but the Tuohy needle was too short to reach the epidural space, so consequently the Tuohy needle was inserted into the T2–3 epidural space assisted by both a fluoroscopy and CT-epidurography (Figs. 3, 4, 5). Autologous blood (18.5 ml) was slowly injected into the T2–3 epidural space. The patient’s headache and dizziness improved greatly after EBP, and he was also able to both stand and walk around without any obvious signs of discomfort. After 16 months of follow-up, the patient’s symptoms had not relapsed.

## Discussion and conclusions

Iatrogenic trauma of the dura at T7–8 and T8–9 was initially suspected because fluid leakage was observed while a Tuohy needle was being advanced at these levels. However, there was no evidence of CSF leakage at T7 to T9 according to the MR images. The leakage location was far from the Tuohy needle puncture levels. During the loss of resistance technique, changes of pressure in the epidural space and an induced dural tear might have explained the cause of the accident, as Thomas and Thanthulage described [2]. Congenital or acquired dural weakness has been reported as a predisposing factor for SIH [5]. Patients with a fragile dura would be more vulnerable to epidural pressure changes. However, subtle leakage may not cause any symptoms unless the CSF secretion and absorption were in a state of imbalance [1].

The normal dura strength varies from 0.4 to 1.1 kg/mm^2^ [6]. Klumpner et al. demonstrated that pressure elevations at the epidural space can vary according to the delivery speed of saline [7]. The peak pressure reached a level of 0.6 kg/mm^2^, while the delivery speed was 6.7 ml/min. Goeller et al. also demonstrated marked caudal epidural space pressure elevation associated with the bolus injection of a local anesthetic [8]. SIH after an uncomplicated lumbar epidural injection has also been reported [2, 3]. In our patient, several attempts to enter the epidural space with a needle caused us to push much air into the epidural space, which may have exacerbated the changes in epidural pressure.

Based on this case, distinguishing SIH from PDPH based on a patient’s subtle medical history may not be as accurate as we had thought. In this patient, the CSF dripped intermittently from the needle, rather than spurting out continuously as is the normal experience after a dural puncture by an 18-gauge Tuohy needle. Thus, if an atypical manifestation occurred due to an epidural catheterization, MR imaging (or any type of advanced imaging) would be a reasonable option to provide additional information regarding the true cause of the manifestation, including neoplastic or non-neoplastic lesions [9–13]. The changes in CSF pressure may be observed as dural sinus engorgement or papilledema on MR images [14]. MRM is a noninvasive method with a high sensitivity (up to 94%) for demonstrating CSF leakage [15], and it could be used to clarify CSF signals to detect the pooling of CSF leakage. Chen et al. proposed a classification of the appearance of CSF on thin-slice axial MRM images [4]. The classification is based on CSF expansion length compared with the thecal sac by thin-slice axial multiplanar reconstruction MRM. Type A lesions are defined by a lack of CSF expansion, type B lesions are defined by triangular expansion, and type C lesions are defined by high-signal-intensity stripes with lengths less than the width of the thecal sac. Our patient’s CSF leak was graded as a type C, which suggests probable leakage. When the findings of MRM are confirmed positive, clinical treatment with EBP is a reasonable option.

Smith first recommended non-targeted EBP at the lumbar area, as the location was less risky than the thoracic or cervical areas [16]. However, a favorable efficacy of targeted EBP was reported by both Wang et al. and Cho et al. [17, 18]. The number of times in which EBP treatment is attempted could be reduced through the use of the targeted option. In our case, it was difficult to distinguish the epidural space from the intrathecal space without the help of radiographs. With the CT guidance, we were able to avoid placing the EBP injection in the wrong location.

The benefits of radiological imaging in this case included confirming the correct diagnosis, helping place the EBP injection into the epidural space, and ultimately increasing the likelihood of success of EBP.

## Data Availability

Not applicable.

## Abbreviations

CSF: Cerebrospinal fluid
CT: Computed tomography
EBP: Epidural blood patch
MR: Magnetic resonance
MRM: Magnetic resonance myelography
PDPH: Post-dural puncture headache
SIH: Spontaneous intracranial hypotension
